# Real-Time, Semi-Automated Fluorescent Measurement of the Airway Surface Liquid pH of Primary Human Airway Epithelial Cells

**DOI:** 10.3791/59815

**Published:** 2019-06-13

**Authors:** Vinciane Saint-Criq, Iram J. Haq, Aaron I. Gardner, James P. Garnett, Christopher Ward, Malcolm Brodlie, Michael A. Gray

**Affiliations:** 1 Epithelial Research Group, Institute for Cell and Molecular Biosciences, Faculty of Medical Sciences, Newcastle University; 2 Respiratory Group, Institute of Cellular Medicine, Faculty of Medical Sciences, Newcastle University; 3 Paediatric Respiratory Medicine, Great North Children's Hospital, Newcastle upon Tyne Hospitals NHS Foundation Trust; 4 Boehringer Ingelheim Pharma GmbH & Co

**Keywords:** Biochemistry, Issue 148, Airway surface liquid, acid-base balance, pH, plate-reader, air-liquid interface, airway epithelium, cystic fibrosis

## Abstract

In recent years, the importance of mucosal surface pH in the airways has been highlighted by its ability to regulate airway surface liquid (ASL) hydration, mucus viscosity and activity of antimicrobial peptides, key parameters involved in innate defense of the lungs. This is of primary relevance in the field of chronic respiratory diseases such as cystic fibrosis (CF) where these parameters are dysregulated. While different groups have studied ASL pH both in vivo and in vitro, their methods report a relatively wide range of ASL pH values and even contradictory findings regarding any pH differences between non-CF and CF cells. Furthermore, their protocols do not always provide enough details in order to ensure reproducibility, most are low throughput and require expensive equipment or specialized knowledge to implement, making them difficult to establish in most labs. Here we describe a semi-automated fluorescent plate reader assay that enables the real-time measurement of ASL pH under thin film conditions that more closely resemble the in vivo situation. This technique allows for stable measurements for many hours from multiple airway cultures simultaneously and, importantly, dynamic changes in ASL pH in response to agonists and inhibitors can be monitored. To achieve this, the ASL of fully differentiated primary human airway epithelial cells (hAECs) are stained overnight with a pH-sensitive dye in order to allow for the reabsorption of the excess fluid to ensure thin film conditions. After fluorescence is monitored in the presence or absence of agonists, pH calibration is performed in situ to correct for volume and dye concentration. The method described provides the required controls to make stable and reproducible ASL pH measurements, which ultimately could be used as a drug discovery platform for personalized medicine, as well as adapted to other epithelial tissues and experimental conditions, such as inflammatory and/or host-pathogen models.

## Introduction

The airway epithelium is covered by a thin (~10 μm) fluid layer termed the airway surface liquid (ASL). The composition and depth (hydration) of this ASL is tightly regulated and controls the efficiency of airway clearance by the mucociliary escalator^1,2,3,4^. In recent years, the importance of the ASL H^+^/HCO_3_^-^ content has been demonstrated by different groups due to its ability to regulate ASL hydration^5^, airway inflammation^6^ and infection^7,8^ as well as mucus viscosity^8,9^. Importantly, although there exists some controversies, many studies have reported dysregulation of the airway pH in chronic airway diseases such as asthma^10,11,12^, COPD^11^, bronchiectasis^11^, chronic rhinosinusitis^13,14^ and cystic fibrosis (CF)^5,9,15,16,17^, which suggests that therapies that restore ASL pH could be useful to treat multiple types of chronic airway diseases. CF is the most common autosomal recessive genetic disease in Caucasian populations and is due to mutations in the CF transmembrane conductance regulator (CFTR) gene. This gene codes an anion (HCO_3_^-^ and Cl^-^) channel that plays a crucial role in ion and fluid transport and homeostasis across epithelia^18^. Although CF is a multi-organ disease, the lung pathology is the main cause of morbidity and mortality^19,20^ and considering the primary defect in CF is an impaired transport of Cl^-^ and HCO_3_^-^, one can hypothesize that extracellular fluid pH in people with CF will be dysregulated compared to people who do not have CF. Thus, the measurement of ASL pH has been a topical area of CF research and different groups have developed techniques to measure ASL pH in CF airways.

In vivo, airway pH has been measured using different techniques, from micro-probes (fiber-optic, gold or mobidium probes)^5,21,22,23,24^ to pH measurements of expectorated material or exhaled breath condensate (EBC)^10,11,12,25,26,27^. In the research field of CF, pH is being widely studied due to its potential clinical implications. Theoretically, making the airways more alkaline could increase bacterial killing and improve mucociliary clearance and airway homeostasis as a whole. However, in vivo/ex vivo studies report a wide range of pH values, and to date, results are not conclusive regarding the existence of a difference in pH between non-CF and CF airways. In the early 2000s, different groups reported the pH of the EBC. In non-diseased groups, pH values ranged from 4.6 to 8.5 but interestingly, EBC pH was found more acidic during exacerbations in people with CF^12,27^. More recently, in vivo measurements of the ASL in human and animal models of CF have reported conflicting results^16,17,21,22,23,24^ and it is still unclear if CF airways are more acidic than non-CF airways.

As in vivo measurement of the lower ASL pH has proven difficult due to the very small amount of fluid lining the airways and potential presence of mucus plugs in disease, many groups have turned to in vitro experiments to measure ASL pH, mainly using three different methodologies. The first approach uses dextran-coupled cell-impermeant pH-sensitive fluorescent dyes which are added as a dry powder, either directly to the ASL or by using an inert fluid called perfluorocarbon (PFC)^5,8,16,17,28,29,30,31,32^. However, this technique provides little control over the exact quantity of dye that is added to the cultures and presents a risk of dye aggregates and big differences in concentration between samples and/or experiments and even within the same sample. It has also generally been performed with a confocal microscope, which limits its applicability and in many cases, prevents detailed monitoring of multiple samples and changes in recording conditions. The second method employed to measure ASL pH is the use of pH-sensitive microelectrodes^5,15^. ASL pH measurements are therefore not dependent on fluorescent dye concentration and should give more robust and reproducible results. However, this method does not allow for dynamic, real-time measurements of ASL pH, nor is it easy to make multiple readings under different conditions. It is also a labor-intensive, complex, process that requires specialist equipment (microelectrode fabrication/electrophysiological recording devices) and training for collection of the samples for subsequent pH measurement and calibration. Moreover, these two techniques have also shown some inconsistencies in the ability to produce reproducible results: using the pH-sensitive fluorescent dye method, Tang et al. reported values of 7.35 for non-CF ASL and 7.0 for CF ASL^8^ whereas in a more recent paper from the same group, ASL pH was 6.9 and 6.4 for non-CF and CF, respectively^17^. In a similar manner, microelectrode measurements gave values of 6.4 in non-CF ASL and 6.1 in CF ASL in a study from 2003^15^ whereas the same group reported values of 6.7 for non-CF ASL and 6.45 for CF ASL in a study from 2013^5^. Finally, in the third approach, researchers add a relatively large volume of weakly buffered solution onto the apical (mucosal) surface of the cultures, thus destroying thin film conditions and altering ASL composition, and potentially its regulation. pH is then measured either using pH-sensitive fluorescent dyes^33^, by a pH-stat titration method in an Ussing chamber^13,14^, or requires the diluted ASL to be removed from the cultures and pH measured using a pH electrode, analyzer or litmus strips^34^. Another difficulty in the accurate measurement of ASL pH is the establishment of a standard curve that is as precise as possible. Indeed, whether the readings are performed with an electrode that will measure the difference in electrical potential via a resin or using pH-sensitive fluorescent dyes, both these approaches will be affected by the local microenvironment of the samples being measured. More specifically, the dissociation constant (Kd) of the dyes may vary considerably depending on the temperature, ionic strength, viscosity as well as potential interactions of the dye with cellular constituents such as proteins and potentially mucus.

In order to try and overcome many of these technical issues, as well as to develop a more dynamic, simpler and higher throughput method, we have established an in vitro technique that records ASL pH in primary hAEC cultures using a cell-impermeant pH-sensitive fluorescent dye in a standard commercial plate-reader. The method generates reproducible, dynamic, semi-automated, real-time measurements of the ASL pH of fully differentiated 3D cell cultures under thin film conditions. Through the use of a multiple-well plate reader, this semi-automated assay can make near simultaneous measurements of pH for up to 24 conditions over 12 h and can monitor the effect of adding various agonists or inhibitors. In this paper we describe the methodology in detail and report representative results under positive and negative control conditions that validates the technique.

## Protocol

Primary non-CF (n = 3 donors, age 34, 27 and 23 years old) and CF (n = 3 donors, all F580del/F508del; age 40, 41, unknown) hAECs were a kind gift from Dr. Scott H. Randell (Marsico Lung Institute, The University of North Carolina at Chapel Hill, United States) and were obtained under protocol #03-1396 approved by the University of North Carolina at Chapel Hill Biomedical Institutional Review Board. The cells were grown according to previously published methods using the growth and differentiation media described by Fulcher and Randell^35,36^.

### 1 Sample preparation

1. Grow primary hAECs on 6.5 mm diameter semi-permeable supports (**Table of Materials**) at air-liquid interface for at least 28 days, as previously described^35,36^.
2. Prepare 50 mL of sterile solution of HCO_3_^-^ containing Krebs buffer solution (HCO_3_^-^ KRB, concentrations are given in mM NaHCO_3_ (25) NaCl (115), KCl (5), CaCl_2_ (1), MgCl_2_ (1), D-glucose(5)) and filter-sterilize using a 0.2 μm syringe filter.
3. Change the basolateral medium to fresh differentiation medium as described in 1.1^35,36^.
  NOTE: It has been shown that basolateral glucose concentration affects ASL pH^33^. At this stage, the glucose content of the basolateral compartment can be controlled by replacing the medium by buffered solutions of known glucose concentrations.
4. Wash the apical surface of the cells by adding 150 µL of HCO_3_^-^ KRB and incubate for 20 min at 37 °C, 5% CO_2_.
5. Remove the apical wash without disrupting the epithelium by aspirating it carefully using a sterile glass Pasteur pipet and a sterile P200 pipet tip linked to an aspiration pump that creates a vacuum in the collection bottle. At this stage, there should be as little liquid remaining on the apical surface as possible to restore the air-liquid interface.
6. Incubate the cells for a further 30 min at 37 °C, 5% CO_2_.

### 2 Background measurement

1. Turn on the plate reader and the computer.
2. Open the dashboard.
3. Click on **Spark 10M**, open the temperature control and set to 37 °C. Open the gas control and set the CO_2_ to 5%.
4. Wait until temperature and CO_2_ have reached their targets.
5. Open the plate reader drawer, insert the humidity cassette filled with 6 mL of dH_2_O on each side. Ensure the lid and bottom of the plate are clean – if not, clean with 70% of ethanol on a piece of tissue - and place the plate in the humidity cassette.
  NOTE: Throughout the experiment, the tissue culture plate lid is kept on the plate and only removed when adding drugs or changing the basolateral medium, which are performed in a tissue culture laminar flow hood to keep the cultures in a sterile environment.
6. Open the Spark Method editor and set up the parameters on the software as follows:
  1. Select the appropriate plate template (for 6.5 mm diameter semi-permeable supports, select the 24 well plate) and the wells that will be monitored during this experiment.
  2. Add a temperature and CO_2_ control panel and set them to 37 °C and 5%, respectively. Tick the **wait for temperature/gas** boxes.
  3. Add a kinetic loop panel and select the Duration as the Loop type and set it to 5-10 min. Choose Not Defined as the Interval type to enable continuous reading.
    NOTE: The timing of continuous reading depends on the number of wells/conditions. 5 min is long enough for 6-12 wells whereas a full plate containing 24 conditions will require 10 min of continuous measurements.
  4. Within the kinetic loop, add two “Fluorescence intensity” panels, using the drag and drop function, that will be set up for the pH-sensitive and the pH insensitive fluorescent dyes respectively. Set excitation and emission wavelengths to 560 and 590 nm, respectively, for the pH-sensitive dye and 495 and 520 nm, respectively, for the pH-insensitive dye.
  5. Set the number of flashes to 30 and the z-position to 33200 for each fluorophore.
    NOTE: The z-position and gain settings are dependent on the characteristics of the plate reader. Set the gain manually to a value that will give high enough counts so that differences between samples will be picked up but low enough so that the addition of an agonist will not generate values out of the range of detection.
  6. Set the multiple read per well to user defined as a circle type of 3 × 3 size with a border of 4750 µm.
7. Click the start button to make a background measurement and OK to confirm the lid of the humidity cassette is in place.
8. At the end of the measurement, open the plate reader drawer, take the plate out and Pplace the cellsit back in the incubator while preparing the fluorescent dye mix solution.
9. Prepare the fluorescent dye mix solution by adding 2 µL of 1 mg/mL dextran-coupled pH-sensitive (pHsens) fluorescent dye to 0.2 µL of 10 mg/mL dextran-coupled pH-insensitive (pHins) fluorescent dye and 0.8 µL of sterile HCO_3_^-^ KRB for a final volume of 3 µL per condition.
  NOTE: The total volume of dye mix solution should be prepared for n wells + 1 if there are between 1 and 10 samples, or n wells + 2 if there are between 11 and 24 samples. Dextran-coupled dyes are reconstituted in filtered-sterile HCO_3_^-^ KRB solution, aliquoted and stored at -20 °C. Any chemical can be added at this stage for a 16-24 h incubation period^37^ on the apical surface. Chemicals should be prepared as 0.1x, as the final volume, after absorption of the excess fluid by the culture, will be around 0.3 µL for a 6.5 mm diameter semi-permeable support.
10. Carefully add 3 µL of dye mix (see 2.8) to the apical surface of the cells and incubate overnight at 37 °C, 5% CO_2_.

### 3 Kinetic measurement

1. Repeat steps 2.1 to 2.4 to prepare the plate reader.
2. Click on the Open icon and select the method file used for the background measurements
3. In the kinetic loop panel, keep the loop type as Duration and set it to 08 hours. Change the Interval type to Fixed and set it to 5 minutes. Keep the Fluorescence Intensity panels the same as for the background measurements
  NOTE: Interval type for background measurements is set to “not defined” in order to allow continuous reading. For kinetic as well and calibration experiments, the interval type is set to “fixed” with an interval of 5 min. This can be adjusted according to the design of the experiment and the number of conditions.
4. Open the plate reader drawer; insert the humidity cassette filled with 6 mL of dH_2_O on each side. Ensure the lid and bottom of the plate are clean – if not, clean with 70% ethanol on a piece of tissue - and place the plate in the humidity cassette, with its cover.
5. Start fluorescence readings by clicking on **Start**. Click **OK** after ensuring the lid of the humidity cassette is in place.
6. After n cycles, usually between to 12 and 24, which is equivalent to 1 to 2 hours, click **Pause** to interrupt the experiment. Take the plate out and apply any drugs/agonists basolaterally to the different samples.
  NOTE: When the cells are taken out of the plate reader, CO_2_ escapes and this will induce an increase in ASL pH as shown by a drop in pH-sensitive dye fluorescence. This CO_2_-induced pH change reverses within 10-15 min after placing the cultures back in the plate reader.
7. Put the plate back in the humidity cassette on the tray, reposition the humidity cassette lid and click **Continue** in order to further record ASL pH and monitor the effect of the drugs/agonists on ASL pH.

### 4 In situ pH calibration

1. Take the plate out of the plate reader.
2. Aspirate the basolateral medium/solution.
3. Add 750 µL and 1 µL of highly buffered standard curve solutions to the basolateral compartment and apical surface, respectively.
  NOTE: Highly buffered standard curve solutions contain (in mM) NaCl (86), KCl (5), CaCl_2_ (1.2), MgCl_2_ (1.2), NaHEPES or MES or Tris (100 mM). Use MES to buffer solutions with a pH lower than 7, NaHEPES for solutions of pH 7-7.5 and Tris for solution with pH 8. Clamp the pH to the desired value using HCl.
4. Switch the CO_2_ off on the plate reader or set it to 0.1% and place the plate back in the humidity cassette.
5. Set up the plate reader with the same parameters as described previously but with no CO_2_ as in step 3.2.
6. Start fluorescence readings, every 5 min for 1-1.5 h.

### 5 Evaluation of the effect of dye concentration and suspension volume on calibration data

1. Prepare enough pH-sensitive and insensitive dye mixture to record the fluorescence at a minimum of 4 different pH values in 3 different volumes.
  NOTE: Here, mix 1 was prepared with 26 µL of pH-sensitive (1 mg/mL) and 2.6 µL of pH-insensitive (10 mg/mL) and mix 2 with 13 µL of pH-sensitive (1 mg/mL) and 1.3 µL of pH-insensitive (10 mg/mL).
2. Distribute 2.2 µL or 1.1 µL of mix 1 or mix 2, respectively, into 12 wells of a 96 well plate and add enough calibration solutions to obtain final volumes of 50, 100 or 200 µL and mix well.
  NOTE: In this set up, fluorescence counts will be recorded for concentrations of dyes of 5 µg/mL (in 200 µL), 10 µg/mL (in 100 or 200 µL), 20 µg/mL (in 50 or 100 µL) or 40 µg/mL (in 50 µL).
3. Turn the plate reader on, set the temperature to 37 °C and insert the plate in the plate reader. Do not turn the CO_2_ controller on.
  NOTE: As this is a short experiment and only requires enough time to equilibrate the temperature, the humidity cassette is not required.
4. Adjust the z-position and gain for the 96 well plate and use the same parameters as for the experiment done on semi-permeable supports.

### 6 Data analysis

1. Save all data to spreadsheets and create a new file.
2. In the background file, select all mean data for each sample/condition for both wavelengths, copy and paste to the new file. Calculate the mean background for each well and each wavelength.
3. Repeat this with the calibration and kinetic data and subtract the background from each data point for each wavelength.
4. For each time point and every sample, calculate the ratio between pH-sensitive and pH-insensitive fluorescence
5. If all the samples were obtained from an individual donor, calculate the mean of the ratios at each time point of the calibration curve
  NOTE: It is important to generate as many calibration curves as donors or basolateral solutions. Indeed, these parameters can affect the background readings or the rate of absorption of the fluid, which in turn will affect the dye concentration and therefore the calculated pH.
6. For each time point, generate a standard curve from the ratios, plotting the known pH values on the x-axis and the ratios on the y-axis.
7. Determine the time point at which ratios are stable, fit a linear regression line and obtain the equation for this line.
8. From the kinetic data, calculate the pH for each time point and plot the pH on the y-axis and the time on the x-axis
  NOTE: Resting/basal pH can be calculated by averaging data points over the stable measurement of pH before addition of any agonist or any other intervention. The effect of an agonist can be characterized by calculating the difference in pH before and after (a certain amount of time) the treatment or by fitting a non-linear curve to the data points directly after the intervention. This will give additional information about the t_1/2_ and the maximal value. Finally, the rates of acidification or alkalinization can also be obtained from the slope of a straight line fitted to the first points after the intervention.

## Representative Results

**Figure 1:**
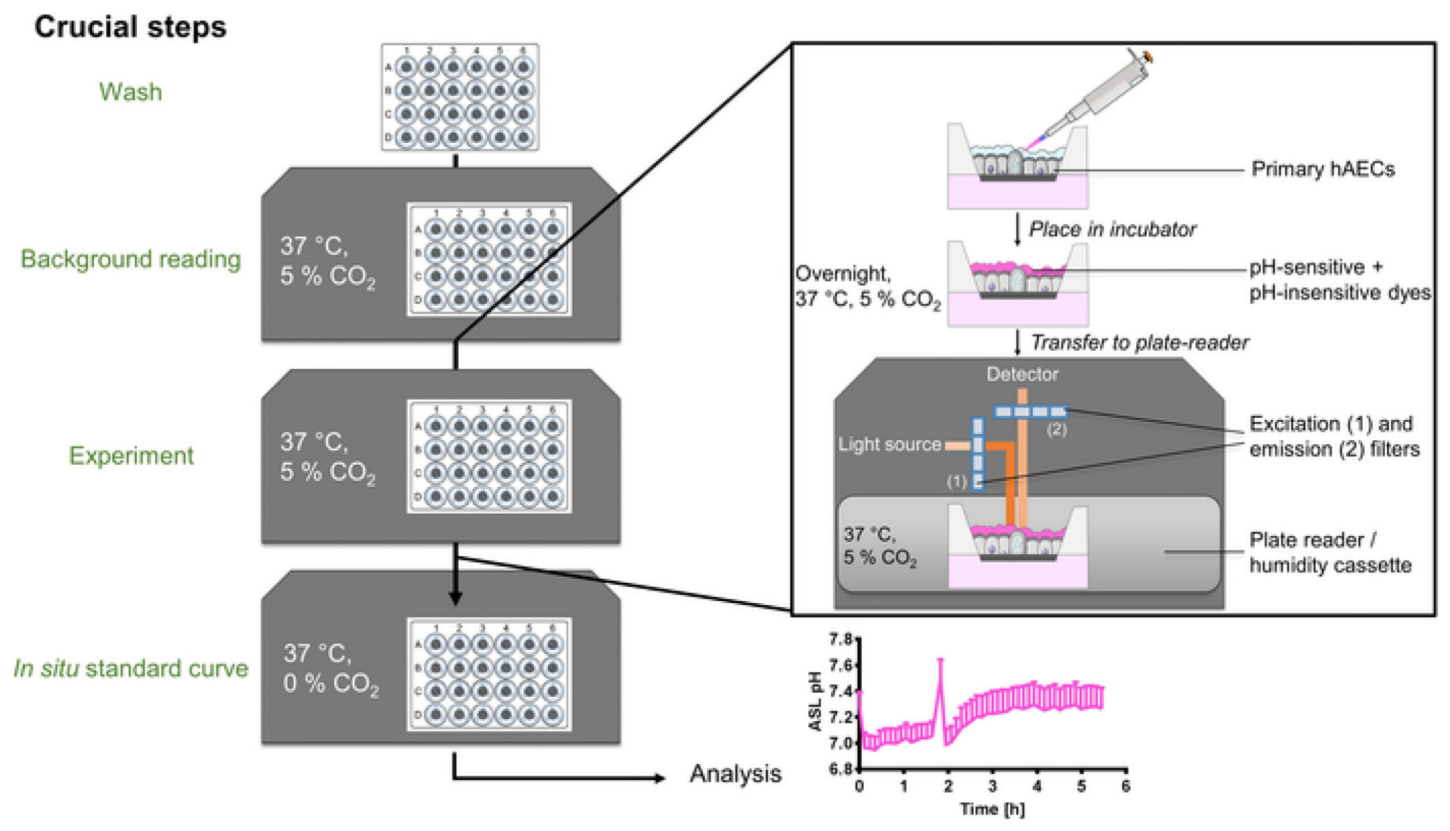
Schematic of the ASL pH measurement method. After washing the cultures and performing a background reading, primary human airway epithelial cells (hAECs) ASL are loaded with dextran coupled pH-sensitive and pH-insensitive dye mixture overnight at 37 °C, 5% CO_2_. The following day, the plate is transferred to a temperature and CO_2_-controlled plate reader and fluorescence from both dyes is recorded over time. After the experiment, an in situ calibration is performed and data analyzed and presented as ASL pH over time.

The technique described above enables the dynamic measurement of ASL pH in up to 24 separate primary hAECs cultures. Figure 1 shows a schematic of the main steps and equipment set up. The overnight-loaded cells are placed in a CO_2_ and temperature controlled plate reader in which fluorescence from dextran-coupled pH-sensitive and pH-insensitive dyes are recorded every 5 min.

**Figure 2:**
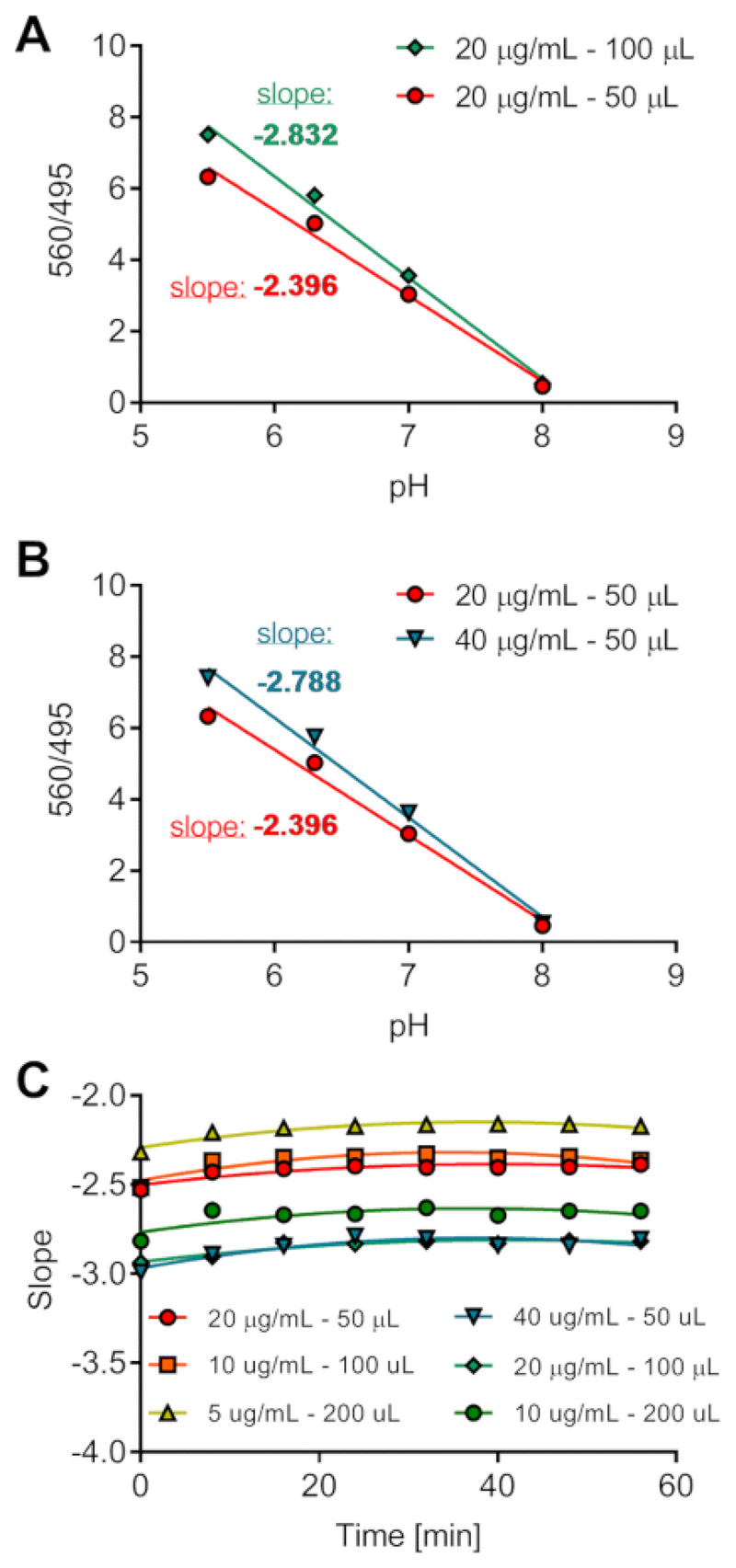
Optimization of the pH calibration in vitro. Different volumes of solutions of known pH and containing different dye concentrations were loaded onto a 96 well plate and fluorescence was recorded over 1 h. Effect of volume (**A**) and dye concentration (**B**) on fluorescence ratios. Ratios were plotted against pH for time-point 24 min. (**C**) The slope of change in fluorescence was calculated for each solution and plotted as a function of time (in min).

**Figure 3:**
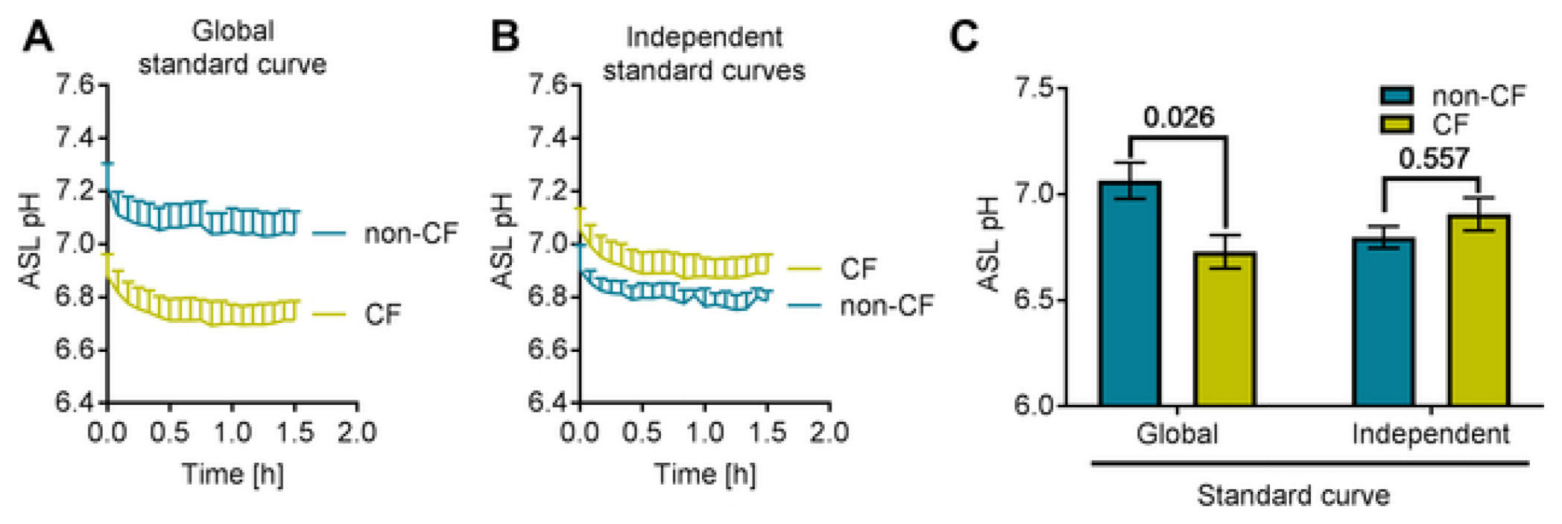
Optimization of the analysis of the pH calibration on primary hAECs in situ. (**A**) Representative traces of ASL pH obtained from a single standard curve averaging data from non-CF and CF cultures. (**B**) Representative traces of ASL pH obtained from independent standard curve performed on non-CF or CF cultures. Each data set was calculated from its own calibration curve. (**C**) Evaluation of the differences in ASL pH between non-CF and CF cultures as a function of how the calibration was performed. Data represent the mean ± SEM from n=3 experiments, 2-way ANOVA, Sidak's multiple comparisons test).

First, we investigated the effect of different volumes and dye concentrations on the fluorescence counts and therefore on the 560/495 ratio. Indeed, the purpose of adding the pH-insensitive to the pH-sensitive dye is to correct for the variability in ASL loading. However, it was important to test this assumption and evaluate if we could use a standard calibration curve performed in the absence of cells in a 96 well plate for all the experiments and cell types. We monitored fluorescence counts over 1 h in 50, 100 or 200 µl of calibration solutions (at pH 5.5, 6.5, 7 or 8) containing 5, 10, 20 or 40 µg/mL of dyes. The results are presented in Figure 2A-C, and show that for the same pH and the same concentration of dyes, the reported pHsens/pHins emission ratio (560/495 on the y-axis) differed depending on the volume (Figure 2A). Additionally, at the same pH and same volume, different dye concentrations provide different ratio values (Figure 2B). Therefore, changes in volume or dye concentration will affect the absolute value of pH calculated from the emission ratio. Figure 2C shows that the time required for temperature equilibration is approximately 15-20 min. To confirm the effect of dye concentration and volume on emission ratios, we recorded fluorescence from dyes loaded in the ASL of primary non-CF and CF hAECs in situ. We then performed the calibration and analyzed the results by (1) generating one global standard curve from all the samples or (2) generating two independent standard curves for each cell type (non-CF and CF). ASL pH from both cell types were then plotted against time (Figure 3A,B) and averaged (Figure 3C). ASL pH values obtained from a single global standard curve showed a significant difference between non-CF and CF cultures (Figure 3A,C) whereas ASL pH was not significantly different between CF and non-CF hAECs when pH was calculated from independent standard curves (Figure 3B,C). These results show the importance of generating independent calibration curves for each experiment and within experiment, for each donor sample, since when the calibration curves were averaged together, higher pHsens/pHins ratio values were found in CF cultures, indicating a more acidic pH (Figure 3C).

**Figure 4:**
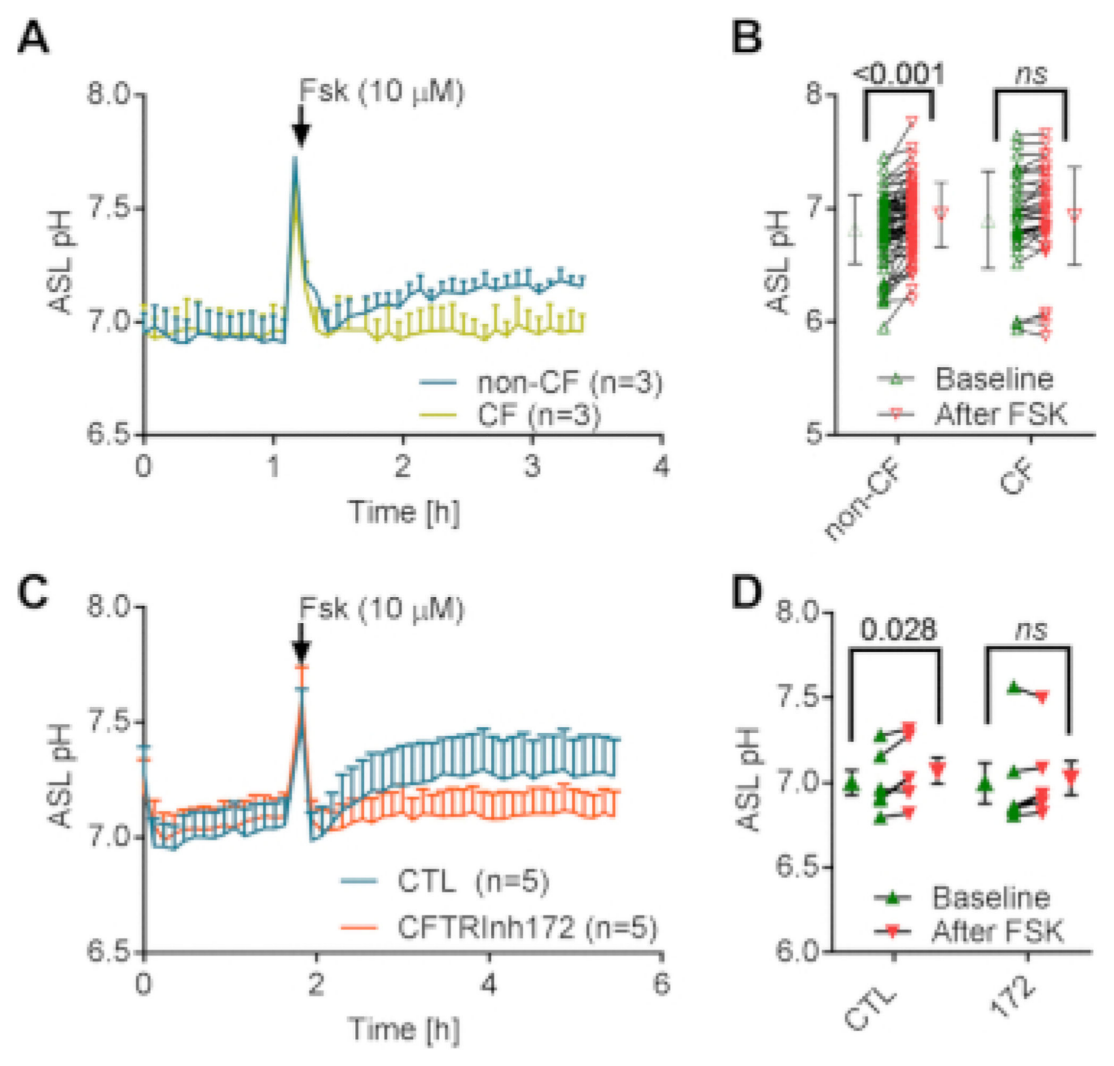
Dynamic ASL pH measurement in response to CFTR activation by forskolin. (**A**) Representative traces of the effect of forskolin (Fsk, 10 µM) on the kinetics of ASL pH over time in non-CF and CF hAECs. Data represent the mean ± SEM from n=3 experiments. (**B**) Summary of the effect of Fsk on ASL pH in non-CF and CF cultures. Data represent the mean ± SD from n=69 non-CF cultures and 35 CF cultures (2-way ANOVA, Sidak's multiple comparisons test). (**C**) Representative traces of the effect of CFTR_inh172_ (172, 20 µM) on the Fsk-induced increase in ASL pH in non-CF hAECs. Data represent the mean ± SEM from n=5 experiments. (**D**) Summary of the effect of 172 on Fsk-induced alkalinisation of the ASL in non-CF cultures. Data represent the mean ± SEM from n=5 experiments (2-way ANOVA, Sidak's multiple comparisons test).

In order to further validate our technique, we then required a positive control to demonstrate that the technique was capable of detecting an ‘expected’ change in ASL pH. As the presence of a more acidic ASL in CF cells is still controversial, we used the cAMP agonist forskolin, as a positive control condition, to stimulate HCO_3_^-^ secretion through CFTR. Expected results would show a forskolin-induced alkalinisation of the ASL in non-CF cells that would be largely decreased or abolished in CF cells depending on the severity of the mutations. Figure 4A shows representative traces of ASL pH of non-CF and CF cells over time and Figure 4B shows the mean data of ASL pH before and after treatment with forskolin in both cell types. We can obtain different information from these results. First, as already shown in Figure 3B,C, the resting ASL pH was not different between non-CF and CF epithelia. Second, the first 3-4 time-points after pausing the experiment to treat the cells with forskolin, showed a large increase in pH that recovered within ~15 min. This was due to the drop in CO_2_ concentration between the plate reader (5%) and the tissue culture safety cabinet (~0%). According to the Henderson Hasselbalch equation, a pH of 7 in a 5% CO_2_ environment equates to a concentration of HCO_3_^-^ of ~9.3 mM. When the cells are removed from the plate reader, a drop in CO_2_ concentration to 0% will theoretically lead to an increase in pH of >8. Figure 4A shows that ASL pH increased to ~7.8 which can be explained by the lapse of time repositioning the plate in the plate reader (i.e., in a 5% CO_2_ environment). Finally, as predicted, addition of basolateral 10 µM forskolin (Fsk) significantly increased ASL pH in non-CF cultures only. As it has been shown by different groups that there exists a difference in steady-state ASL pH between CF and non-CF epithelia, we wanted to further investigate the apparent absence of a pH difference in our experiments and the role of CFTR. To do this we pre-incubated non-CF cultures with the specific CFTR inhibitor, CFTR_inh172_ (172). As stated in the protocol section 2.8, the dye mix was prepared as stated above and the inhibitor was added at a concentration of 0.1X = 2 µM. According to the literature, ASL height of non-CF cells is approximately 10 µm. In a semi-permeable support of 6.5 mm diameter, the theoretical volume of the ASL is therefore π × 3.25^2^ = 0.3 µL. By adding 3 µL of dye + 172 at 2 µM, the concentration of the inhibitor, after absorption of the excess fluid, will theoretically be 20 µM (1x, desired concentration). Representative traces in Figure 4C and mean summary in Figure 4D show that 172 did not reduce resting ASL pH but did prevent the forskolin-induced increase in ASL pH, thus confirming our results obtained from non-CF *versus* CF cultures and further validating our technique.

**Figure 5:**
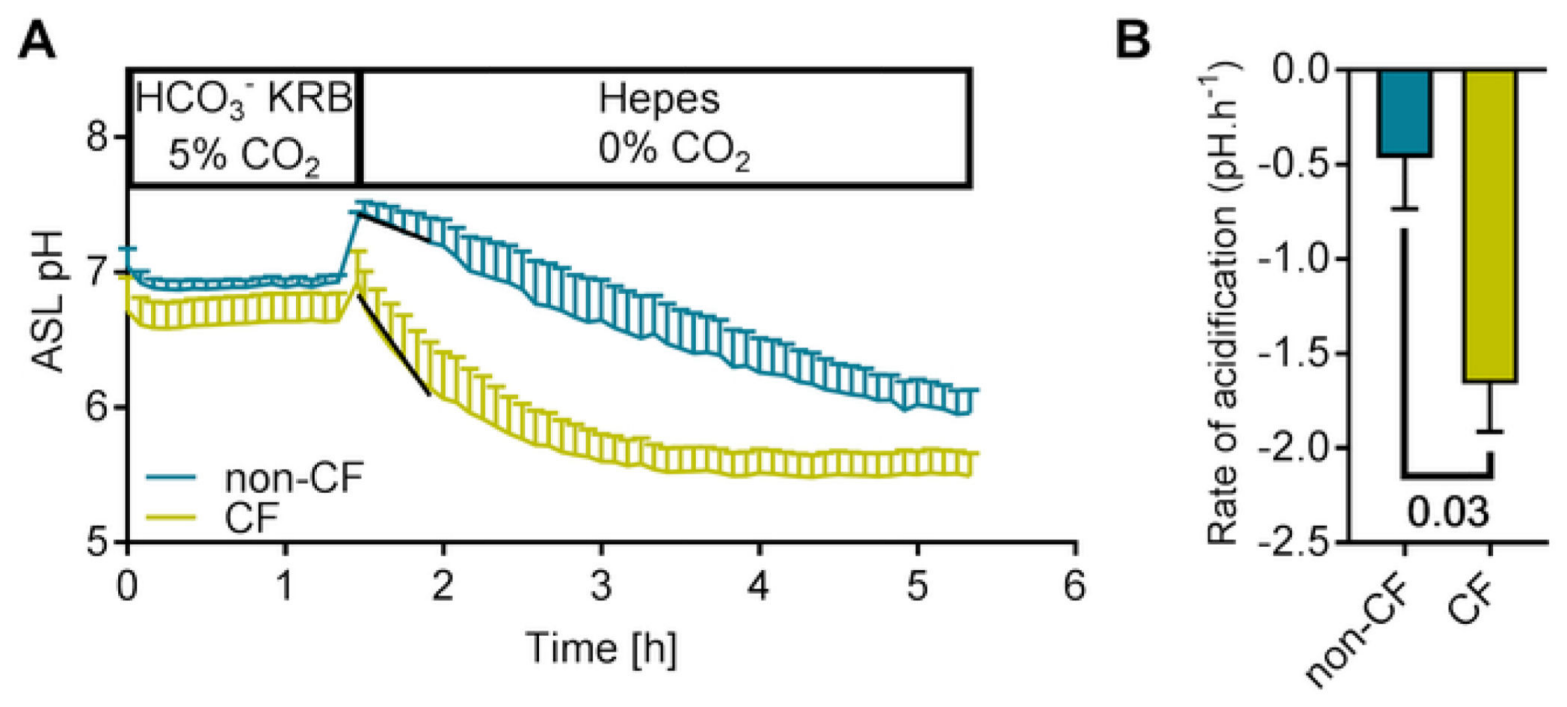
Dynamic changes in ASL pH in response to HCO_3_^-^ removal. (**A**) Representative traces showing the effect of HCO_3_^-^ removal on the kinetics of ASL pH over time in non-CF and CF hAECs. The initial rates of acidification were obtained via the slope of a straight line fitted to 7 time-points after HCO_3_^-^ removal. Data represent the means ± SEM from n=6 and 7 experiments on non-CF and CF cultures respectively. (**B**) Summary of the initial rates of acidification following HCO_3_^-^ removal. Data represent the means ± SEM from n=6 and 7 experiments on non-CF and CF cultures respectively (Mann-Whitney test).

Finally, as stated in the protocol section 6.8, rates of acidification/alkalinization can be calculated by fitting a linear regression to the initial time points after the intervention. Figure 5A shows that removing the basolateral HCO_3_^-^ containing solution (HCO_3_^-^ KRB) and replacing it with a HEPES buffered solution, in the absence of CO_2_, induced a marked acidification of the ASL. This is consistent with the lack of HCO_3_^-^ inhibiting transepithelial HCO_3_^-^ secretion, which allows constitutive proton secretion by these airway cells to steadily reduce ASL pH^15,17^ Interestingly, the initial rate of acidification of non-CF cells was significantly slower than CF cultures (Figure 5B).

## Discussion

Here we provide a detailed protocol for the dynamic measurement of ASL pH in primary human airway epithelial cells. Critical steps include washing the mucus off the apical surface of the cells, measuring and subtracting the background using the same parameters as in the experiment, optimizing the z-position and gain and performing an in situ pH calibration.

The first step of washing the cells is crucial as a thick layer of mucus might (i) prevent the dyes from reaching the periciliary layer (PCL) and (ii) delay or prevent the detection of changes in fluorescence in response to agonists/inhibitors. Our method was developed to study how primary hAECs modulated the activity of HCO_3_^-^ and H^+^ transporters in response to agonists. While it will be interesting to investigate how changes in PCL pH relate to changes in mucus pH, further development of this protocol is needed, including the use of different molecular weight-dextrans to differentially target the 2 layers and z-scans through the whole ASL.

Background measurement is another important step of this protocol. The apical surface of fully differentiated primary airway epithelia is rarely completely flat which will affect the light path and therefore the background. Ensuring that the background readings are performed in the same local points of the wells as during the experiment is critical for reproducibility and stability of the recordings.

Optimizing the z-position and gain are necessary steps that need to be set up for each different concentration of fluorescent dye that will be used. This will prevent high inter-experiment variability. Once set up, our assay provides stable and reproducible results. One of the reasons for this is that the dyes are added on the apical surface on the cells in a small volume of fluid that is easily reabsorbed by the epithelium, leaving a homogenously labeled ASL. Another method to stain the ASL, that can be equally successful, used dry powder or a “suspension” in PFC. Although this might be time-saving (as the experiments are usually performed within 2 h), it is unlikely that the dry dyes fully solubilize in the ASL and thus might form clumps. Thus different concentrations of pH-sensitive dye will be found over the surface of the epithelial cells.

The in situ pH calibration is an important step in order to obtain accurate, reproducible results. As shown and explained in the results section, differences in ASL volumes will affect the fluorescence counts and therefore the interpolated pH values (Figure 2 and Figure 3). Whilst different groups have previously published ASL pH measurements, a wide range of values have been obtained even between different studies published by the same group^8,17^. We believe that by performing in situ calibrations, results will become more reproducible. Compared to other pH calibration techniques, which use the high K^+^/nigericin (or multiple ionophores) method to generate the standard curve^28,29,30^, the assay presented here has the advantage that, as long as every step is performed in a safety cabinet, the cells used for ASL pH can be washed, kept and reused for other experiments provided that the treatments performed do not irreversibly affect the epithelial cells.

The development and optimization of this assay has provided reproducible results and we believe this method will help other groups with their ASL pH measurement. However, this technique has also some limitations due to set up and the type of cells that are being used. Monitoring ASL pH over a longer time period than that presented here (>8-10 h) might prove difficult as a long-term high humidity environment might damage the equipment and the fact that most plate readers only offer the option to record kinetic readings over a certain amount of time (typically 24 h). The use of fully differentiated primary hAECs is crucial in the way that different stages of differentiation will affect the expression of HCO_3_^-^ and H^+^ transporters. However, there is virtually no possibility to precisely control the volume of ASL in cells grown under thin film conditions. As stated in the protocol and results sections, changes in volume will affect the fluorescence ratio and it is unfortunately necessary to assume that in cells grown from a single individual, seeded on the same day on different semi-permeable supports, ASL volumes will be the same. Arising from this limitation, any agonist or inhibitor that will affect fluid secretion or absorption will affect the ASL volume and presumably the fluorescence ratios. However, in our assay, the calibration curve is performed at the end of the experiment, so we can presume that these changes in volume will affect the calibration ratios in the same way as during the kinetic experiment. For this reason we advise groups that would be interested in developing this assay, to use at least 2-3 replicates per condition tested as this will allow for the establishment of a standard curve for each condition.

Here we present a simple, semi-automated, assay that allows real-time measurement of mucosal surface pH under thin-film conditions. It has the capacity of investigating dynamic pH responses in many cultures in a near-simultaneous way that allows inter and intra-donor comparisons. Upscaling this method to a 96 well plate format using polarized system (HTS 96 well plates)^38^ would provide even higher throughput as a drug discovery assay. Moreover, we have shown how this technique can be used to study the acute effect of agonists on ASL pH and we have already published that this method can be used to study the long-term effect of an apical proton pump inhibitor on CF hAECs ASL^39^. As pH has been shown to regulate infection, inflammation, mucus viscosity and ion transport, identifying molecular targets that can increase pH will be valuable in the research fields of chronic lung diseases and this technique will potentially facilitate the development of drug screening in personalized medicine approaches. Finally, since dysregulation in acid-base homeostasis plays a major role in other diseases, this protocol can be adapted, with optimization steps, to different equipment (plate readers) and cell types, such as other epithelial cells. Extracellular acidity is a characteristic of cancer^40,41,42^ and this assay could help determine how solid tumors produce low pH_e_ or could be used as a low-throughput drug screening assay for restoration of pH homeostasis. Similarly, as for chronic airway diseases, it could also provide a platform for development of a personalized medicine approach.
